# Le pneumothorax spontané comme une manifestation évolutive de la polyarthrite rhumatoide: à propos d'une observation clinique et revue de la litterature

**DOI:** 10.11604/pamj.2015.22.319.7888

**Published:** 2015-12-02

**Authors:** Magaye Gaye, Assane Ndiaye, Mouhamed Lamine Fall, Souleymane Diatta, Papa Adama Dieng, Papa Salmane Ba, Amadou Gabriel Ciss, Mouhamadou Ndiaye

**Affiliations:** 1 Service de Chirurgie Thoracique et Cardiovasculaire, Chnu de Fann BP 5035; 2 Service d'Anesthésie et de Réanimation, Chnu de Fann BP 5035

**Keywords:** Pneumothorax, polyarthrite rhumatoïde, méthotrexate, Pneumothorax, rheumatoid arthritis, methotrexate

## Abstract

La polyarthrite rhumatoïde est une maladie systémique inflammatoire caractérisée par une destruction des synoviales articulaires et des lésions systémiques extra articulaires. Les nodules pulmonaires font partie de ces dernières. Leur évolution peut aboutir à un pneumothorax spontané. Nous rapportons le cas d'un adulte jeune au long passé de polyarthrite rhumatoïde qui a présenté deux épisodes de pneumothorax spontané. Il était admis dans notre service, en urgence, pour un pneumothorax droit spontané et massif sur terrain de polyarthrite rhumatoïde au stade de déformation. Il était sous méthotrexate. La radiographie standard du thorax et la tomodensitométrie montraient un décollement pleural complet droit, des nodules et des images excavées sur les deux champs pulmonaires. Il a bénéficié d'un drainage thoracique aspiratif permettant une bonne ré-expansion pulmonaire. Le pneumothorax spontané constitue une manifestation rare des lésions pulmonaire de la polyarthrite rhumatoïde. Il s'agit le plus souvent d'une manifestation évolutive de la maladie mais aussi une circonstance de découverte de cette dernière. L'implication des immunosuppresseurs reste à être prouvée. Ainsi la polyarthrite rhumatoïde doit être considérée dans la recherche étiologique d'un pneumothorax spontané.

## Introduction

La polyarthrite rhumatoïde est une maladie inflammatoire du système caractérisée par une destruction des synoviales articulaires et des lésions systémiques extra articulaires. Les nodules pulmonaires font partie de ces dernières. Leur évolution peut aboutir à un pneumothorax spontané. Nous rapportons le cas d'un adulte jeune au long passé de polyarthrite rhumatoïde qui a présenté deux épisodes de pneumothorax spontané.

## Patient et observation

**Figure 1:**
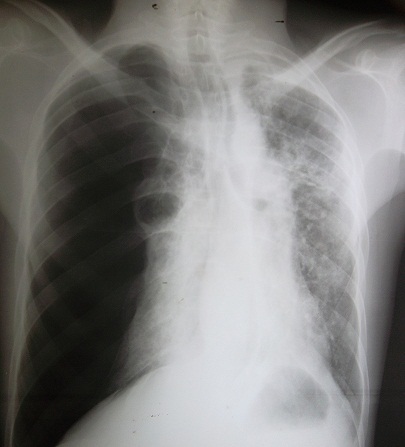
Pneumothorax complet droit avec un nodule excavé

**Figure 2:**
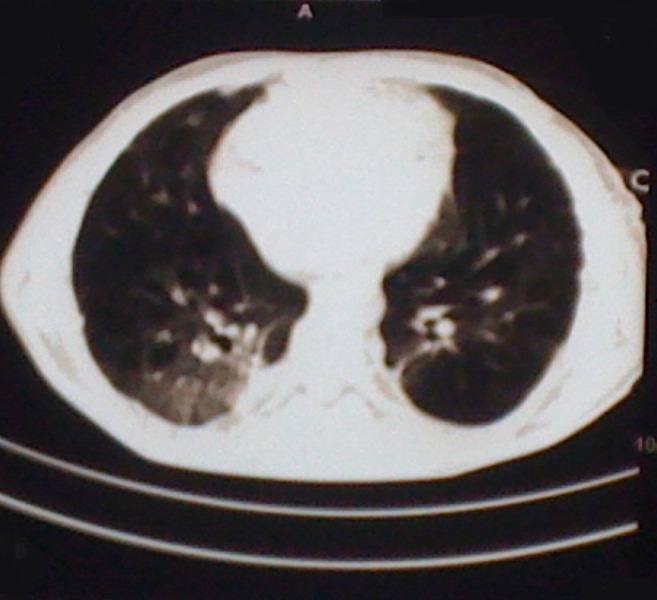
Après mise en place du drain thoracique

**Figure 3:**
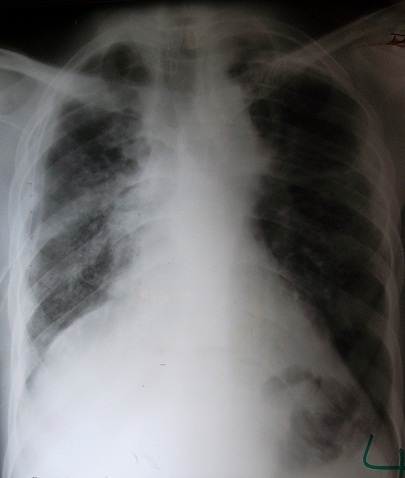
Mise en évidence de nodules excavés dans le parenchyme pulmonaire droit

**Figure 4:**
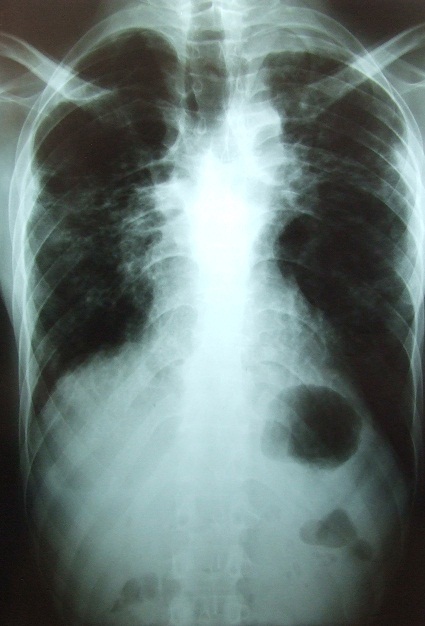
Radiographie de contrôle après ablation du drain

C. D. âgé de 26 ans, était admis dans notre service, en urgence, pour un pneumothorax droit spontané et massif sur terrain de polyarthrite rhumatoïde mal suivie au stade de déformation. Il était sous méthotrexate avec notion d'arrêt du traitement sans avis médical. Dans ses antécédents, on ne retrouvait pas de tabagisme, ni d'exposition professionnelle ou de tuberculose pulmonaire. Il avait présenté un épisode de pneumothorax spontané gauche, trois mois auparavant. L'examen retrouvait un syndrome d’épanchement gazeux droit avec tirage et battements des ailes du nez. Par ailleurs, on notait des déformations, des phalanges des doigts très invalidantes, en coup de vent. La radiographie du thorax montrait un décollement pleural complet avec des cavités sous pleurales (Figure 1) et la tomodensitométrie thoracique objectivait des nodules excavés sur les deux champs pulmonaires (Figure 2). Il a bénéficié d'un drainage pleural aspiratif (Figure 3) associé à un traitement antalgique. Une kinésithérapie respiratoire avait complété le traitement. Le contrôle radiographique, au septième jour, montrait un poumon qui était revenu à la paroi avec une poche apicale résiduelle (Figure 4) autorisant l'ablation du drain. Il est sorti de l'hôpital, au huitième jour, et est ré-adressé à son rhumatologue, pour poursuite de son traitement immunosuppresseur.

## Discussion

Le pneumothorax spontané est très rare, dans la polyarthrite rhumatoïde. Il est secondaire à la présence des nodules rhumatoïdes pulmonaires. Ces derniers constituent une des manifestations pulmonaires rares de la polyarthrite rhumatoïde [1]. Les autres sont la pleurésie, les infections pulmonaires, les artérites et l'hypertension pulmonaire, les bronchectasies et le syndrome de Caplan. Ils sont souvent associés aux nodules sous cutanés et leur localisation est souvent pleurale et ou scissurale [2]. Leur évolution se fait vers la régression ou l'augmentation de volume et la fistulisation par nécrobiose. Si cette dernière se produit au niveau d'une bronche, sa manifestation clinique sera une hémoptysie, et si elle se fait dans la cavité pleurale, on assiste à un pneumothorax [2]. La survenue du pneumothorax est considérée comme l’évolution naturelle des nodules pulmonaires mais d'autres incriminent les médicaments immunosuppresseurs utilisés contre la polyarthrite rhumatoïde, à savoir, la méthotrexate, le flunomide, infliximat et étanercept [3, 4]. Le pneumothorax peut être observé chez un patient suivi pour une polyarthrite rhumatoïde [1] ou être la circonstance de découverte de cette maladie; précédant, ainsi, les lésions articulaires [2]. Les cas de pneumothorax spontané sur polyarthrite rhumatoïde décrits dans la littérature concernent surtout les sujets âgés de plus de 40ans [1–5], contrairement à notre patient qui en a moins. La biologie peut montrer une coïncidence avec une éosinophilie et une accélération de la vitesse de sédimentation par contre la positivité du facteur rhumatoïde n'est pas toujours observée [1, 2, 4, 5]. La surveillance radiologique permet de suivre l’évolution de ces nodules pulmonaires et permet de faire le diagnostic différentiel avec les carcinomes primaires et métastatiques pulmonaires, la tuberculose, les infections fongiques et les embolies septiques [1, 5]. L'histologie de ses nodules reste la seule certitude diagnostique, en montrant une lésion par hyperplasie lymphocytaire interstitielle avec des cellules géantes multinuclées [1, 2, 4]. Le traitement de première intention, de ce pneumothorax, est le drainage pleural aspiratif; mais en fonction des lésions associées d'autres moyens peuvent être utilisé comme la pleurodèse par la chirurgie thoracique vidéo assistée et la bullectomie [1, 2, 4, 5].

## Conclusion

Le pneumothorax spontané constitue une manifestation rare des lésions pulmonaires de la polyarthrite rhumatoïde. Il peut en être une manifestation évolutive ou une circonstance de découverte. La polyarthrite rhumatoïde est une affection qu'il faut savoir évoquer d'autant plus qu'il existe un pneumothorax spontané et des manifestations articulaires.
